# Worth the Wait? Comparison of Emergency Department Patients’ Waiting Room Tolerance for Real Patient Care vs Training/Simulation Scenarios

**DOI:** 10.5811/westjem.48916

**Published:** 2026-04-14

**Authors:** Alice Rogan, Euan Watt, Stephanie Murphy, Emily Wheeler, Lisa Woods, Sagar Galwankar, Brad Peckler

**Affiliations:** * Wellington Hospital, Department of Emergency Medicine, Wellington, New Zealand; † Florida State University, College of Medicine, Department of Emergency Medicine, Sarasota, Florida; ‡ Victoria University, School of Mathematics and Statistics, Wellington, New Zealand

## Abstract

**Introduction:**

In-situ simulation offers a realistic training environment with a higher level of fidelity compared to other simulation models. It is associated with enhanced knowledge retention and a higher level of composure during real clinical encounters. One common barrier to undertaking in-situ simulation is the concern that it contributes to a delay in providing patient care. In this study we gave patients in the waiting room seven hypothetical emergency medical scenarios, two of which were training simulation scenarios, and we asked them how long they would be willing to delay their care if the different scenarios were actually occurring in the emergency department (ED). Our objective was to investigate whether patients in the ED waiting room would be willing to delay their care if they knew that there were simulation training scenarios occurring.

**Methods:**

This was a prospective convenience sample of participants conducted at a Level 1 trauma centre. Participants completed a survey that presented seven hypothetical scenarios, including two in-situ simulation scenarios. They were then asked to indicate the amount of additional wait time they would deem acceptable for each scenario.

**Results:**

Responses to the two in-situ simulation scenarios indicated that 342 (40%) and 335 (40.5%) of the 827 study participants, respectively, were willing to wait > 40 minutes for these to occur. In contrast, and after controlling for age, sex, waiting time, and time of recruitment, subjects reported they would tolerate shorter wait times for simulation scenarios than for real patient-care scenarios. [Willingness to wait > 40 minutes for the five real scenarios ranged from 70.5–79.9%, P < .05).

**Conclusion:**

While patients demonstrated lower tolerance for simulation-related delays than for routine clinical care, our results showed that most were still willing to wait up to an additional hour to allow in-situ simulation to proceed. These findings indicate that in-situ simulation is broadly acceptable to patients and supports its continued use in clinical settings.

## INTRODUCTION

Simulation training has long been recognised as a vital component of undergraduate and postgraduate medical education.^1^–^3^ The benefits of this learning modality are vast, including improved technical and nontechnical skills.^4^–^8^ Simulation is a highly effective way of consolidating theoretical knowledge by provoking a physiological response that enhances retention of data^9^ but also allows learners to practice and refine the technical skills that cannot be learned by reading a book.^10^–^11^ Furthermore, there is substantial evidence to support the role of simulation training in developing the non-technical skills essential for high-functioning teams, such as expert communication, managing interpersonal conflict, and allocating effective roles.^12^,^13^ Simulation is also an effective way to refine approaches to high-acuity, low-occurrence presentations, improve interdisciplinary team cohesion, and identify crucial system issues that can be improved for future real-life scenarios.^14^,^15^

In-situ simulation refers to simulated clinical scenarios in participants’ usual working environment. This realistic training environment offers a higher level of fidelity compared to other settings, which has been shown to enhance knowledge retention^16^,^17^ and help learners maintain a higher level of composure during subsequent real clinical encounters. It is an unrivalled means of providing genuine simulation training. It is beneficial for simulated scenarios involving interdisciplinary team members who may benefit from exposure to emergency department (ED) design and equipment.^18^,^19^

One common barrier to undertaking in-situ simulation is the concern that it contributes to a delay in providing patient care.^20^ As ED access block and waiting times continue to increase globally,^21^–^23^ there is potential for in-situ simulation training to reduce timely service provision for patients. While there is research examining patients’ attitudes toward the length of time they are willing to wait to be seen,^20^,^24^ data are sparse on whether patients are willing to tolerate longer wait times for medical teams to undergo in-situ simulation training. Our objective in this study was to investigate whether patients in the ED waiting room would be willing to delay care if they knew that there were simulation training scenarios occurring.

## METHODS

This was a prospective convenience sample of participants in a Level 2 trauma centre in South Florida with an annual volume of 90,000 with an average of 250 pretentions seen each day. While most patients are seen from the waiting room in approximately four hours, their wait time can be as long as six hours due to the influx of population to the area during the winter months. After patients were triaged and waiting to be seen in the waiting room, they were approached to be involved in the study, and informed written consent was obtained. Ethics approval was obtained from Florida State University ethics board. Participants were > 18 years of age, and English was their primary language. The concept of in-situ simulation was explained to them in lay terms. To avoid bias, participants were not informed that the study’s primary outcome was the wait time for the in-situ simulation scenarios.

**Figure 1:**
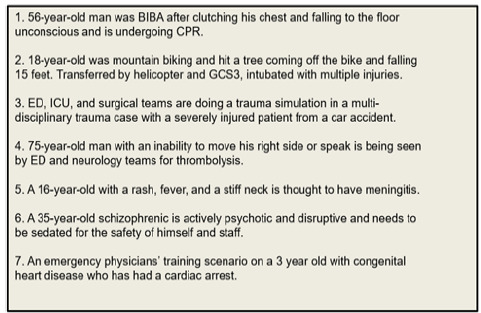
Questions in the survey given to patients in the emergency department waiting room, in a study examining the length of time they would be willing to wait to be seen for an in-situ simulation scenario. *BIBA*, brought in by ambulance; *CPR*, cardiopulmonary arrest; *GCS3*, Glasgow Coma Scale score 3; *ED*, emergency department; *ICU*, intensive care unit.

For each participant, investigators recorded basic demographic information and the time of day they were interviewed (12 am–12 pm, 12 pm–6 pm, and 6 pm–12 am), the length of time they had been waiting at that point, and their total predicted waiting time to be seen. Participants were then given an electronic tablet preloaded with a survey using HIPAA-compliant software (Phase Zero, Boston, MA). The survey presented seven hypothetical scenarios and asked participants to indicate the amount of additional wait time they would deem acceptable for each scenario (Figure 1). Cases were designed and agreed upon by consensus of the emergency clinician investigators based on what was felt to represent common emotive presentations. Each scenario was written in plain language to be understandable to a lay reader. In-situ training was explained during the consent process to clarify for the survey respondents that simulation was staff training. Possible wait times were divided into seven options using minutes: 0–20; 21–40; 41–60; 61–80; 81–100; 101–120; and > 120 minutes. All data were password protected and accessible only accessible by the study investigators. We excluded from analysis articipants with missing data.

**Population Health Research Capsule**

What do we already know about this issue?

*In-situ simulation improves team performance but its use is limited by concerns that it may delay patient’s emergency care and prolong waiting times*.

What was the research question?

*Are ED patients willing to accept longer waits if in-situ simulation training is occurring?*

What was the major finding of the study?

*40–40.5% of patients would wait >40 minutes for simulation vs 70.5–79.9% for real care (P<.05)*.

How does this improve population health?

*Patients tolerated simulation delays less than real care, yet most would still wait up to forty minutes for in-situ simulation to proceed*.

The primary outcome was to compare the amount of time patients were willing to wait for actual treatment vs the simulation-training scenarios. We used mixed-effects models to examine differences in the selected wait times between medical scenarios. We chose this class of models to group responses from the same participant, fitting the participant as a random intercept. The scenario, age, sex, wait time, and time of day were included in the model as fixed effects. Statistical significance was determined as *P* < .05, and we conducted Bonferroni-adjusted pairwise comparisons where appropriate. The analysis used the *Mclogit* package in R v4.3.1 (R Foundation for Statistical Computing, Vienna, Austria) for Windows (Microsoft Corporation, Redmond, WA.^25^,^26^

## RESULTS

We recorded 1,104 initial interactions from 1,069 participants (13 patients participated twice or more). After removing incomplete responses or missing clinical or demographic data, 827 participants remained in the final dataset for analysis. Of these 827 study participants, 443 (53.6%) were female, and 384 (46.4%) were male. The median age was 54 (interquartile range [IQR] 35–70). The time of day in which participants were surveyed was divided into three timeframes for analysis: 12 am–12 pm (13%), 12 pm–6 pm (39%) and 6 pm–12 am (50%). Baseline demographics are displayed in Table 1.

**Figure 2:**
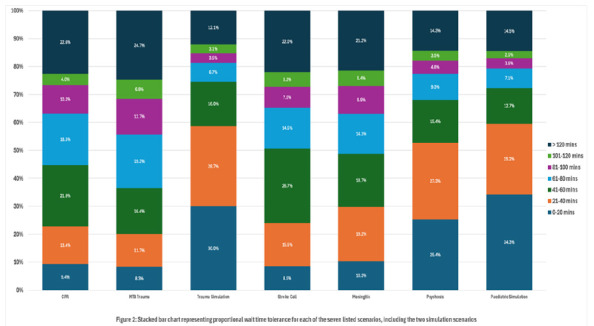
Stacked bar chart representing proportional wait time tolerance for each of the seven listed scenarios, including the two simulation scenarios *CPR*, cardiopulmonary arrest; *MTB, mountain bike*.

A summary of responses indicating tolerable wait time for each of the seven scenarios is shown in Table 2 and displayed as a stacked bar chart in Figure 2. See supplementary tables for the estimated coefficients from the mixed-effects model and pairwise comparisons from which the following results are summarised.

The responses to scenarios 3 and 7, detailing in-situ simulation, indicate that 41.4% and 40.5% of participants were willing to wait more than 40 minutes for these scenarios, respectively. However, after controlling for age, sex, waiting time, and time of day of recruitment, participants tended to tolerate shorter wait times for simulation scenarios than for almost any other medical scenario (all *P* < .05). Respondents were more likely to indicate that a wait time of 0–20 minutes was acceptable for simulation scenarios (Q3 = 41.6%, Q7 =. 49.8%) than for other medical scenarios (ranging from 4.5–34.6%; see Table S2). The percentage of participants who selected an acceptable additional wait time of 0–20 minutes or 21–40 minutes for the two simulation scenarios (Q3 and Q7) was significantly higher (*P* < .001) than any other medical event besides the psychiatric scenario (Q6 vs Q7; *P* > .05). See Supplementary Material for details of the pairwise comparisons. Conversely, participants were significantly more likely to tolerate wait times of 61–80, 81–100, 101–120, and > 120 minutes for the actual medical scenarios (excluding the psychiatric scenario) when compared to the simulation scenarios (all *P* < .05).

Analysis showed that participants who had already been waiting for 0–20 minutes were more likely to tolerate an additional wait time of > 120 minutes than people who had been waiting longer than 20 minutes (all *P* < .001). Those participants submitting a response between midnight and 12 pm were significantly more likely to select an appropriate wait time of 0–20 minutes compared to those submitting a response between 12 pm–6 pm, and between 6.pm to midnight. Those participants submitting a response between 6 pm and midnight were significantly more likely to select an appropriate wait time of 101–120 minutes compared to those submitting a response between midnight and 12 pm, and between 12 pm–6 pm. Females were significantly more likely than males to select an additional acceptable wait of > 120 minutes (*P* < .001).

## DISCUSSION

Despite the clear benefits of in-situ simulation for medical education, there is a real risk that its delivery is curtailed within our current clinical environment due to competing priorities. As health systems issues and access block continue to cause ED waiting times to balloon globally, it is easy to see these planned education sessions being cancelled to allow the redeployment of staff to direct clinical care. However, this would have far-reaching consequences for undergraduate and postgraduate medical education, as well as patient safety and quality of care. Just as simulation teaching brings some benefits that cannot be harnessed from any other teaching method, in-situ simulation is crucial for identifying systems issues that would otherwise go undiscovered. This could have a dramatic impact on future patients treated in the same institution if they are not discovered during simulation teaching.

In-situ training was explicitly explained during the consent process in lay language to clarify for the survey respondents that simulation was in fact staff training. To our knowledge, this is the first study to explore the attitudes of actual ED patients towards in-situ simulation, which affects their wait time for medical care. Until now, we had assumed that we are doing waiting room patients a disservice if we cause slight delays in their care by undertaking in-situ simulation teaching. Yet we had not asked the patients themselves. Whilst truly emergent or life-threatening presentations should always take absolute priority, the data from this study suggest that some delay in being seen is tolerated for most patients with less emergent issues.

Patients in this study showed a lower tolerance for delays caused by simulation than for real clinical care, yet many were still willing to wait up to an extra hour for in-situ simulation to proceed. Comparable international data are scarce; however, a New Zealand qualitative study found that ED patients were generally willing to wait an additional 20–30 minutes, and potentially longer, depending on clinical urgency.^27^ Most patients are unfamiliar with simulation but become more accepting once it is clearly explained. Transparent communication helps minimise dissatisfaction related to extended waits.^27^

A full in-situ scenario and structured debrief can be completed within 30–60 minutes, with the clinical space required only for the scenario itself. Debriefing can easily occur in a non-clinical area, avoiding further impact on patient flow. For most departments, these findings support implementing regular in-situ simulation with sufficient duration to deliver meaningful educational value while remaining acceptable to most patients.

Other notable findings from this study include that, unsurprisingly, the data showed a general trend for patients to tolerate longer additional wait times if they had only been waiting for a short period when surveyed. Interestingly, patients were significantly less likely to wait for the psychiatric scenario than for the medical scenario. This may or may not represent inherent biases within the population and requires further exploration.

## LIMITATIONS

There were several limitations to this study. Firstly, as we used convenience sampling methods and data were not collected for those that declined to participate, this may have introduced selection bias. Furthermore, most responses were received in the evening or overnight hours, which could have skewed the results. Therefore, additional work could examine a more balanced sample across all hours of the day to ensure that the results are replicated. Secondly, this study employed a single-centre design in a tertiary Level 1 ED in South Florida. Therefore, caution should be exercised when assuming that attitudes towards delays in care will be similar in other countries or, indeed, in EDs in other regions of the state. Despite using lay language, in-situ simulation training may have been misunderstood by survey participants.

Finally, the severity of each respondent’s presenting illness or pain score was not accounted for by triage score or any other means. Patients triaged and in the waiting room would presumably have a lower triage score. We thought self-perception of severity would be challenging to interpret and skew responses. Minimal clinical characteristics and patient factors were collected, and this may limit interpretation and generalisability of patient responses.

## CONCLUSION

Patients demonstrated lower tolerance for simulation-related delays than for routine clinical care, yet most were still willing to wait up to an additional hour to allow in-situ simulation to proceed. These findings indicate that in-situ simulation is broadly acceptable to patients and supports its continued use in clinical settings. If replicated in further studies, these findings could provide a clear mandate to continue valuable in-situ simulations despite the growing access block in EDs.

## Supplementary Information



## Figures and Tables

**Table 1:** Baseline demographics of survey respondents and wait time when the survey was administered as part of a study examining the length of time they would be willing to wait to be seen for an in-situ simulation scenario.

Variable	Respondents N=827
Age, Median (IQR)	54 (35–70)
Sex, n (%)	
Female	443 (53.6%)
Male	384 (46.4%)
Wait time, n (%)	
0–20 min	197 (23.8%)
21–40 min	255 (30.8%)
41–60 min	144 (17.4%)
61–80 min	75 (9.1%)
81–100 min	37 (4.5%)
101–120 min	32 (3.9%)
> 120 min	87 (10.5%)

*IQR*, interquartile range.

**Table 2:** Summary of times patients were willing to wait for each scenario.

Question	Response (N=827)						

	(1) 0 – 20 minutes	(2) 21 – 40 minutes	(3) 41–60 minutes	(4) 61–80 minutes	(5) 81–100 minutes	(6) 101–120 minutes	(7) >120 minutes
Q1: A 56-year old man was brought in by an ambulance after clutching his chest and falling to the floor unconscious and is undergoing CPR.	78 (9.4%)	111 (13.4%)	181 (21.9%)	153 (18.5%)	84 (10.2%)	33 (4.0%)	187 (22.6%)
Q2: An 18 year old female was mountain biking and hit a tree coming off the bike and falling 15 feet. She was brought in by helicopter and is unconscious on a breathing tube with obvious head, abdominal and upper leg injuries. The trauma surgeons and ED physicians are trying to save her.	69 (8.3%)	97 (11.7%)	136 (16.4%)	160 (19.3%)	105 (12.7%)	56 (6.8%)	204 (24.7%)
Q3: The ED, intensive care, and trauma surgeons are doing a trauma simulation and practicing teamwork, skills training, and medical expertise in a multi-disciplinary trauma case with a severely injured patient from a car accident.	248 (30.0%)	237 (28.7%)	132 (16.0%)	55 (6.7%)	29 (3.5%)	26 (3.1%)	100 (12.1%)
Q4: 75 year old man presented ot the hospital with an inability to move his right side or speak. It is thought he is having a stroke (brain attack) and is being seen by the ED physicians and the neurologists to determine if he can be given a medication to try to reverse the stroke by breaking up the clot.	70 (8.5%)	128 (15.5%)	221 (26.7%)	120 (14.5%)	62 (7.5%)	44 (5.3%)	182 (22.0%)
Q5: A 16 year old girl is being seen with a rash and a fever and a stiff neck. She is thought to have meningitis and is being treated agressively by the ED physicians	85 (10.3%)	159 (19.2%)	163 (19.7%)	118 (14.3%)	82 (9.9%)	45 (5.4%)	175 (21.2%)
Q6: A 35 year old man with known schizophrenia is actively psychotic and disruptive and needs to be sedated for the safety of himself and staff.	210 (25.4%)	226 (27.3%)	127 (15.4%)	77 (9.3%)	40 (4.8%)	29 (3.5%)	118 (14.3%)
Q7: The ED physicians are doing a training scenario on a simulated patient that is a 3-year-old with congenital heart disease that has had a cardiac arrest.	284 (34.3%)	208 (25.2%)	105 (12.7%)	59 (7.1%)	30 (3.6%)	21 (2.5%)	120 (14.5%)

*CPR*, cardiopulmonary arrest; *ED*, emergency department.
