# Supplementary material for: Worth the Wait? Comparison of Emergency Department Patients’ Waiting Room Tolerance for Real Patient Care vs Training/Simulation Scenarios
Source: West J Emerg Med. 2026 Apr 14;27(3):644–50. doi: 10.5811/westjem.48916 (PMC13249442; doi:10.5811/westjem.48916)
Supplement: Supplementary file 1 [file wjem-27-644-s001.docx]

**Supplementary Table 1.** Coefficient estimates from the model fitted

The estimated coefficients for the baseline category logit model for multinomial responses (with a random effect) are reported below as estimate (standard error), and statistical significance indicated by *** = p < .001; ** = p < .01; * = p < .05. This model incorporates 5789 responses from 827 participants.

|  | Additional wait time they would deem acceptable | | | | | |
| --- | --- | --- | --- | --- | --- | --- |
|  | ’21-40mins’ vs ’0-20mins’ | ’41-60mins’ vs ’0-20mins’ | ’61-80mins’vs ’0-20mins’ | ’81-100mins’ vs ’0-20mins’ | ’81-100mins’ vs ’0-20mins’ | ’>120mins’ vs ’0-20mins’ |
| (Intercept) | 0.426  (0.400) | 2.075***  (0.444) | 2.678***  (0.497) | 1.988***  (0.562) | 0.860  (0.642) | 3.829***  (0.618) |
| Question: Q2 vs Q1 | 0.017  (0.245) | -0.082  (0.243) | 0.398  (0.252) | 0.708*  (0.276) | 1.156***  (0.334) | 0.748**  (0.282) |
| Question: Q3 vs Q1 | -0.930***  (0.202) | -2.703***  (0.217) | -3.959***  (0.256) | -4.364***  (0.309) | -3.655***  (0.358) | -4.690***  (0.284) |
| Question: Q4 vs Q1 | 0.303  (0.240) | 0.34  (0.236) | -0.227  (0.257) | -0.324  (0.291) | 0.346  (0.345) | -0.055  (0.287) |
| Question: Q5 vs Q1 | 0.236  (0.229) | -0.381  (0.233) | -0.629*  (0.251) | -0.372  (0.277) | 0.009  (0.339) | -0.512  (0.282) |
| Question: Q6 vs Q1 | -0.749***  (0.205) | -2.417***  (0.219) | -3.167***  (0.245) | -3.511***  (0.291) | -2.999***  (0.351) | -3.847***  (0.279) |
| Question: Q7 vs Q1 | -1.298***  (0.203) | -3.187***  (0.221) | -4.084***  (0.253) | -4.486***  (0.306) | -4.027***  (0.372) | -4.420***  (0.277) |
| Age (years) | -0.007  (0.004) | -0.011*  (0.005) | -0.011*  (0.006) | -0.007  (0.007) | -0.011  (0.007) | -0.007  (0.008) |
| Sex: Male vs Female | -0.016  (0.178) | -0.193  (0.203) | -0.246  (0.233) | -0.336  (0.263) | -0.363  (0.291) | -0.938**  (0.305) |
| Wait time: ’21-40mins’ vs ’0-20mins’ | 0.173  (0.250) | 0.209  (0.283) | -0.282  (0.316) | -0.324  (0.359) | -0.063  (0.389) | -1.098**  (0.403) |
| Wait time: ’41-60mins’ vs ’0-20mins’ | 0.376  (0.285) | 0.216  (0.325) | -0.770*  (0.371) | -0.785  (0.421) | -0.842  (0.464) | -1.780***  (0.480) |
| Wait time: ’61-80mins’ vs ’0-20mins’ | 0.332  (0.349) | 0.347  (0.396) | -0.290  (0.448) | -0.459  (0.510) | -1.024  (0.583) | -1.776**  (0.595) |
| Wait time: ’81-100mins’ vs ’0-20mins’ | 0.098  (0.438) | -0.658  (0.516) | -1.683**  (0.604) | -1.561*  (0.677) | -2.249**  (0.807) | -2.777***  (0.789) |
| Wait time: ’101-120mins’ vs ’0-20mins’ | 0.394  (0.450) | -1.161*  (0.557) | -2.165***  (0.656) | -1.259  (0.694) | -2.299**  (0.833) | -3.714***  (0.907) |
| Wait time: >120mins’ vs ’0-20mins’ | 0.091  (0.338) | -0.203  (0.386) | -1.349**  (0.448) | -1.279*  (0.506) | -1.947***  (0.587) | -2.502***  (0.587) |
| TimeofDay2:  >=12pm and <6pm/<12pm | 0.923***  (0.276) | 0.593  (0.315) | 0.345  (0.364) | 0.046  (0.412) | 0.016  (0.481) | -0.902  (0.469) |
| TimeofDay2: >=6pm/<12pm | 0.680**  (0.254) | 0.592*  (0.289) | 0.699*  (0.334) | 0.587  (0.375) | 1.171**  (0.436) | 0.113  (0.422) |

**Supplementary Table 2**

*The table below contains p-values for the Bonferroni-adjusted pairwise comparisons of questions, for Response = ’0-20 minutes’. The observed percentage of respondents selecting 0-20 minutes as an acceptable wait time is shown in brackets for each question.*

|  | Q1  (9.4%) | Q2  (8.3%) | Q3  (30.0%) | Q4  (8.5%) | Q5  (10.3%) | Q6  (25.4%) | Q7  (34.3%) |
| --- | --- | --- | --- | --- | --- | --- | --- |
| Q1  (9.4%) |  | 1.0000 | <.0001*** | 1.0000 | 1.0000 | <.0001*** | <.0001*** |
| Q2  (8.3%) |  |  | <.0001*** | 1.0000 | .1639 | <.0001*** | <.0001*** |
| Q3  (30.0%) |  |  |  | <.0001*** | <.0001*** | .5031 | .1672 |
| Q4  (8.5%) |  |  |  |  | 1.0000 | <.0001*** | <.0001*** |
| Q5  (10.3%) |  |  |  |  |  | <.0001*** | <.0001*** |
| Q6  (25.4%) |  |  |  |  |  |  | <.0001*** |
| Q7  (34.3%) |  |  |  |  |  |  |  |

Significance: *** = p < .001; ** = p < .01; * = p < .05

**Supplementary Table 3**

*The table below contains p-values for the Bonferroni-adjusted pairwise comparisons of questions, for Response = ’21-40 minutes’. The observed percentage of respondents selecting 21-40 minutes as an acceptable wait time is shown in brackets for each question.*

|  | Q1  (13.4%) | Q2  (11.7%) | Q3  (28.7%) | Q4  (15.5%) | Q5  (19.2%) | Q6  (27.3%) | Q7  (25.2%) |
| --- | --- | --- | --- | --- | --- | --- | --- |
| Q1  (13.4%) |  | 1.0000 | <.0001*** | 1.0000 | .0025** | <.0001*** | <.0001*** |
| Q2  (11.7%) |  |  | <.0001*** | .0166* | <.0001*** | <.0001*** | <.0001*** |
| Q3  (28.7%) |  |  |  | <.0001*** | .0003*** | 1.0000 | .8189 |
| Q4  (15.5%) |  |  |  |  | .3300 | <.0001*** | .0001*** |
| Q5  (19.2%) |  |  |  |  |  | .0002*** | .2331 |
| Q6  (27.3%) |  |  |  |  |  |  | .9795 |
| Q7  (25.2%) |  |  |  |  |  |  |  |

Significance: *** = p < .001; ** = p < .01; * = p < .05

**Supplementary Table 4**

*The table below contains p-values for the Bonferroni-adjusted pairwise comparisons of questions, for Response = `41-60 minutes’. The observed percentage of respondents selecting 41-60 minutes as an acceptable wait time is shown in brackets for each question.*

|  | Q1  (21.9%) | Q2  (16.4%) | Q3  (16.0%) | Q4  (26.7%) | Q5  (19.7%) | Q6  (15.4%) | Q7  (12.7%) |
| --- | --- | --- | --- | --- | --- | --- | --- |
| Q1  (21.9%) |  | .0037** | .0001*** | .1211 | 1.0000 | .0005*** | <.0001*** |
| Q2  (16.4%) |  |  | 1.0000 | <.0001*** | .1202 | 1.0000 | .0471* |
| Q3  (16.0%) |  |  |  | <.0001*** | .0009*** | 1.0000 | .7869 |
| Q4  (26.7%) |  |  |  |  | .0047** | <.0001*** | <.0001*** |
| Q5  (19.7%) |  |  |  |  |  | .0067** | <.0001*** |
| Q6  (15.4%) |  |  |  |  |  |  | .1678 |
| Q7  (12.7%) |  |  |  |  |  |  |  |

Significance: *** = p < .001; ** = p < .01; * = p < .05

**Supplementary Table 5**

*The table below contains p-values for the Bonferroni-adjusted pairwise comparisons of questions, for Response = ’61-80 minutes’. The observed percentage of respondents selecting 61-80 minutes as an acceptable wait time is shown in brackets for each question.*

|  | Q1  (18.5%) | Q2  (19.3%) | Q3  (6.7%) | Q4  (14.5%) | Q5  (14.3%) | Q6  (9.3%) | Q7  (7.1%) |
| --- | --- | --- | --- | --- | --- | --- | --- |
| Q1  (18.5%) |  | 1.0000 | <.0001*** | .2902 | .2125 | <.0001*** | <.0001*** |
| Q2  (19.3%) |  |  | <.0001*** | .1745 | .1252 | <.0001*** | <.0001*** |
| Q3  (6.7%) |  |  |  | <.0001*** | <.0001*** | .1107 | 1.0000 |
| Q4  (14.5%) |  |  |  |  | 1.0000 | <.0001*** | <.0001*** |
| Q5  (14.3%) |  |  |  |  |  | <.0001*** | <.0001*** |
| Q6  (9.3%) |  |  |  |  |  |  | .2347 |
| Q7  (7.1%) |  |  |  |  |  |  |  |

Significance: *** = p < .001; ** = p < .01; * = p < .05

**Supplementary Table 5**

*The table below contains p-values for the Bonferroni-adjusted pairwise comparisons of questions, for Response = ’81-100 minutes’. The observed percentage of respondents selecting 81-100 minutes as an acceptable wait time is shown in brackets for each question.*

|  | Q1  (10.2%) | Q2  (12.7%) | Q3  (3.5%) | Q4  (7.5%) | Q5  (9.9%) | Q6  (4.8%) | Q7  (3.6%) |
| --- | --- | --- | --- | --- | --- | --- | --- |
| Q1  (10.2%) |  | .4666 | <.0001*** | .5246 | 1.0000 | <.0001*** | <.0001*** |
| Q2  (12.7%) |  |  | <.0001*** | .0004*** | .1336 | <.0001*** | <.0001*** |
| Q3  (3.5%) |  |  |  | <.0001*** | <.0001*** | .6456 | 1.0000 |
| Q4  (7.5%) |  |  |  |  | 1.0000 | .0010** | <.0001*** |
| Q5  (9.9%) |  |  |  |  |  | <.0001*** | <.0001*** |
| Q6  (4.8%) |  |  |  |  |  |  | .9861 |
| Q7  (3.6%) |  |  |  |  |  |  |  |

Significance: *** = p < .001; ** = p < .01; * = p < .05

**Supplementary Table 6**

*The table below contains p-values for the Bonferroni-adjusted pairwise comparisons of questions, for Response = ’101-120 minutes’. The observed percentage of respondents selecting 101-120 minutes as an acceptable wait time is shown in brackets for each question.*

|  | Q1  (4.0%) | Q2  (6.8%) | Q3  (3.1%) | Q4  (5.3%) | Q5  (5.4%) | Q6  (3.5%) | Q7  (2.5%) |
| --- | --- | --- | --- | --- | --- | --- | --- |
| Q1  (4.0%) |  | .0582 | .0171* | 1.0000 | 1.0000 | .1685 | .0076** |
| Q2  (6.8%) |  |  | <.0001*** | .8439 | 1.0000 | .0001*** | <.0001*** |
| Q3  (3.1%) |  |  |  | .0009*** | .0005*** | 1.0000 | 1.0000 |
| Q4  (5.3%) |  |  |  |  | 1.0000 | .0088** | .0004*** |
| Q5  (5.4%) |  |  |  |  |  | .0048** | .0002*** |
| Q6  (3.5%) |  |  |  |  |  |  | 1.0000 |
| Q7  (2.5%) |  |  |  |  |  |  |  |

Significance: *** = p < .001; ** = p < .01; * = p < .05

**Supplementary Table 7**

*The table below contains p-values for the Bonferroni-adjusted pairwise comparisons of questions, for Response = ’>120 minutes’. The observed percentage of respondents selecting >120 minutes as an acceptable wait time is shown in brackets for each question.*

|  | Q1  (22.6%) | Q2  (24.7%) | Q3  (12.1%) | Q4  (22.0%) | Q5  (21.2%) | Q6  (14.3%) | Q7  (14.5%) |
| --- | --- | --- | --- | --- | --- | --- | --- |
| Q1  (22.6%) |  | .5144 | <.0001*** | 1 | 1 | <.0001*** | <.0001*** |
| Q2  (24.7%) |  |  | <.0001*** | .0880 | .0250* | <.0001*** | <.0001*** |
| Q3  (12.1%) |  |  |  | <.0001*** | <.0001*** | .3089 | 1 |
| Q4  (22.0%) |  |  |  |  | 1 | <.0001*** | <.0001*** |
| Q5  (21.2%) |  |  |  |  |  | <.0001*** | <.0001*** |
| Q6  (14.3%) |  |  |  |  |  |  | 1 |
| Q7  (14.5%) |  |  |  |  |  |  |  |

Significance: *** = p < .001; ** = p < .01; * = p < .05
